# Diagnostic Value of Metagenomic Next-Generation Sequencing for Pulmonary Infection in Intensive Care Unit and Non-Intensive Care Unit Patients

**DOI:** 10.3389/fcimb.2022.929856

**Published:** 2022-08-15

**Authors:** Jing-Jiang Zhou, Wei-Chao Ding, Yan-Cun Liu, Yu-Lei Gao, Lei Xu, Run-Lu Geng, Ying Ye, Yan-Fen Chai

**Affiliations:** ^1^ Department of Emergency Medicine, Tianjin Medical University General Hospital, Tianjin, China; ^2^ Department of Emergency Medicine, Affiliated Hospital of Xuzhou Medical University, Xuzhou, China

**Keywords:** metagenomic next-generation sequencing, intensive care unit, diagnosis, pulmonary infection, immunocompromised patients

## Abstract

**Objective:**

To evaluate the diagnostic performance of metagenomic next-generation sequencing (mNGS) and culture in pathogen detection among intensive care unit (ICU) and non-ICU patients with suspected pulmonary infection.

**Methods:**

In this prospective study, sputum samples were collected from patients with suspected pulmonary infection for 2 consecutive days and then subjected to DNA or RNA sequencing by mNGS or culture; 62 ICU patients and 60 non-ICU patients were admitted. In the end, comparisons were made on the pathogen species identified by mNGS and culture, the overall performance of these two methods in pathogen detection, and the most common pathogens detected by mNGS between the ICU and non-ICU groups.

**Results:**

In DNA and RNA sequencing, the positive rate of pathogen detection reached 96.69% (117/121) and 96.43% (108/112), respectively. In culture tests, the positive rate of the pathogen was 39.34% (48/122), much lower than that of DNA and RNA sequencing. In general, the positive rate of pathogen detection by sputum mNGS was significantly higher than that of sputum culture in the total and non-ICU groups (p < 0.001) but did not show a significant difference when compared to the result of sputum culture in the ICU group (p = 0.08). *Haemophilus* spp., *Candida albicans*, *Enterococcus* spp., and viruses from the mNGS results were excluded before comparing the overall performance of these two methods in pathogen detection. Specifically, among the 10 most common bacteria implied from the mNGS results, significant differences were observed in the number of cases of *Haemophilus parainfluenzae*, *Acinetobacter baumannii*, *Klebsiella pneumoniae*, *Pseudomonas aeruginosa*, *Stenotrophomonas maltophilia*, *Staphylococcus aureus*, and *Enterococcus faecalis* between the ICU and non-ICU groups (p < 0.05).

**Conclusions:**

This study demonstrated the superiority of mNGS over culture in detecting all kinds of pathogen species in sputum samples. These results indicate that mNGS may serve as a valuable tool to identify pathogens, especially for ICU patients who are more susceptible to mixed infections.

## Introduction

Pulmonary infection is one of the most common clinical infections caused by an extremely broad class of pathogens, including bacteria, viruses, fungi, mycoplasma, chlamydia, and/or other pathogenic microorganisms (Magill et al., 2014). It is challenging to identify the pathogens in non-intensive care unit (non-ICU) patients, not to mention ICU patients who tend to be immunocompromised and/or be connected to a ventilator (Li et al., 2020; Geng et al., 2021). Though the culture has been used as the diagnostic gold standard for many bacterial infections, it is time-consuming, and it cannot identify many atypical pathogens and even fails to identify the most abundant species from time to time (Schlaberg et al., 2017). Thus, it is of crucial importance to look for efficient alternatives with a high degree of sensitivity and specificity to speed up the diagnosis process and guide clinicians to the most appropriate treatment. In recent years, unbiased metagenomic next-generation sequencing (mNGS) is emerging as a valuable tool in the diagnosis of infectious diseases, capable of simultaneously detecting almost all species in one sample (Gu et al., 2019). This technique has a higher sensitivity than culture-based methods, especially for pathogen identification in bronchoalveolar lavage fluid (BALF), blood, and sputum. In this study, we aimed to compare the pathogen species identified by mNGS and culture, the overall performance of these two methods in pathogen detection, and the most common pathogens detected by mNGS between the ICU and non-ICU groups.

## Materials and Methods

### Study Patients

A prospective study was conducted to collect sputum from patients with suspected pulmonary infection throughout the hospital who required sputum culture. The following exclusion criteriawere used: i) sputum samples that failed to pass quality control of mNGS (e.g., contain <10 epithelial cells per low-power field and >25 white blood cells per high-power field), ii) incomplete clinical history, and iii) sample leakage and contamination. Baseline data were collected fromthe electronic medical records of the patients, including demographic characteristics, comorbidities, immunosuppressive state, treatment process, prognosis, and whether admitted to ICUs, including ICU, emergency ICU, neurology ICU, coronary care unit, respiratory ICU, neonatal ICU, and pediatric ICU.

### Specimen Collection and Processing

On January 14 and 15, 2021, 139 sputum specimens (including 17 duplicate specimens) were collected from the inpatients with suspected pulmonary infection in the Affiliated Hospital of Xuzhou Medical University. Depending on the patient’s condition, different procedures were taken for sampling. For patients who were awake and cooperative, they were asked to collect sputum in the morning after rinsing their mouths or brushing their teeth. Patients with dentures should remove their dentures and then spit the sputum directly into a sterile, dry, leak-proof container in order to avoid adding saliva or nasopharyngeal secretions to the sputum samples. For patients with artificial airways or in a coma, the sputum samples were collected by applying negative pressure to a disposable suction tube inserted into the artificial airway to the lungs or through the nasal cavity to the glottis. In the end, a total of 122 cases were included, with 62 cases being ICU patients and the rest being non-ICU patients. Ideally, the volume of all specimens should be >1 ml, part of which was kept under strict sterile conditions for culture, while the rest of it was stored in a sterile sealed container and sent to a Vision Medicals Laboratory in Nanjing for mNGS. If the amount of specimen was too small, DNA testing was performed instead.

### Sputum Culture Test

A sputum sample examined microscopically should contain <10 epithelial cells per low-power field and >25 white blood cells per high-power field. Then, these samples were digested and shaken to homogenates, which were then inoculated to the appropriate medium. Cultures were performed using a PROBACT-K auto marking and culture apparatus (DIASE, Wuhan, China) for a maximum period of 5 days, with bacteria identified by VITEK-2 Compact (Merrier, Shanghai, China).

### Metagenomic Next-Generation Sequencing

Initially, human DNA was removed by Benzonase (Qiagen, Valencia, CA, USA) and Tween20 (Sigma, St. Louis, MO, USA). Then, the bacterial cell wall was digested, and DNA extraction was carried out using a QIAamp^®^ UCP Pathogen DNA Kit (Qiagen), followed by DNA fragmentation, adaptor ligation, biomolecular label (barcode) amplification, and on-board sequencing. Total RNA was extracted with a QIAamp^®^ Viral RNA Kit (Qiagen), and ribosomal RNA was removed by a Ribo-Zero rRNA Removal Kit (Illumina, San Diego, CA, USA). cDNA was generated using reverse transcriptase and dNTPs (Thermo Fisher, Waltham, MA, USA). For negative controls, peripheral blood mononuclear cell (PBMC) samples were collected from healthy donors, diluted to a concentration of 10^5^ cells/ml, and processed in parallel with each batch under the same protocol, with sterile deionized water extracted alongside PBMC samples to serve as non-template controls (NTCs).

Nucleic acid detection and sequencing were performed based on the Illumina IDseq platform. Libraries were constructed for the DNA and cDNA samples using a Nextera XT DNA Library Prep Kit (Illumina, San Diego, CA, USA) (Miller et al., 2019). The quality of libraries was evaluated by a Qubit dsDNA HS Assay Kit, followed by a High Sensitivity DNA Kit (Agilent, Santa Clara, CA, USA) on an Agilent 2100 Bioanalyzer. Library pools were then loaded onto an Illumina NextSeq CN500 sequencer for 75 cycles of single-end sequencing to generate approximately 20 million reads for each library.

### Bioinformatics Analysis

Trimmomatic was used to exclude low-quality reads, adapter contamination, duplicate reads, and reads shorter than 50 bp. Low-complexity reads were removed by Kcomplexity with default parameters (Bolger et al., 2014). Human sequence data were identified and excluded by aligning to a human reference genome hg38 in Burrows-Wheeler Aligner software. For selecting representative assembly for microorganisms from the NCBI Nucleotide and Genome databases (NCBI, 2021), a set of criteria similar to the National Center for Biotechnology Information (NCBI) criteria was designed. The pathogen list was completed according to the *Johns Hopkins ABX Guide* (Johns Hopkins ABX Guide, 2021), *Manual of Clinical Microbiology* (MCM, 12th Edition, 2019), and clinical case reports or research articles published in current peer-reviewed journals (Fiorini et al., 2017), with the final database consisting of ~13,000 genomes. To minimize cross-species misalignments among closely related microorganisms, the RPM of microorganisms sharing a genus or family designation was penalized (reduced), if the species or genus appeared in NTCs. A penalty of 5% was set for species (Gu et al., 2021).

### Statistical Analysis

Continuous variables with normal distribution were expressed as mean ± SD, whereas continuous data with non-normal distribution were expressed as median (Q1, Q3). Categorical data were expressed as numbers (percentages). Comparative analyses were conducted by Pearson’s χ^2^ test, McNemar’s chi-square test, ANOVA, Z test, and Mann–Whitney U test. According to the extracted data, a 2 × 2 contingency table was established to calculate sensitivity, specificity, positive predictive value (PPV), and negative predictive value (NPV). The R 4.1.0 software was used for data analysis, and a two-tailed value of p < 0.05 was considered for significant differences.

## Results

### Characteristics of Basic Information on Samples and Patient Conditions

The basic information on 122 patients with suspected pulmonary infection is presented in **Table 1**. Sputum samples collected from these subjects were successfully tested by culture, while the data of DNA sequencing were incomplete due to an operational miss, thus including results of all non-ICU samples but only 61 ICU samples (99.18%). There were even less data on RNA pathogens because RNA sequencing was only performed on 52 ICU samples (83.87%) and 60 non-ICU samples (100%). In this study, 62 ICU patients (50.82%) and 60 non-ICU patients (49.18%) were included, consisting of 77 men and 45 women with a median age of 62.5 years, a median length of stay of 16 days, and a mortality rate of 7%. There were no significant differences between the ICU group and the non-ICU group in age, gender, and mortality rate (p > 0.05 in all), except for the length of stay (p < 0.001). Of all clinical conditions listed in **Table 2**, the most common ones in patients appeared to be a cerebral stroke, coma, solid tumor, diabetes, chronic obstructive pulmonary disease (COPD), and post-chemotherapy.

**Table 1:** Basic information on samples and patients.

Basic information^*^	Total	ICU^#^	Non-ICU^**^	*p-*Value^##^
Sample number, n (%)	122 (100)	62 (50.82)	60 (49.18)	/
Sputum culture, n (%)	122 (100)	62 (50.82)	60 (49.18)	/
DNA sequencing, n (%)	121 (99.18)	61 (98.39)	60 (100)	/
RNA sequencing, n (%)	112 (90.98)	52 (83.87)	60 (100)	/
Age, median years (Q1, Q3)	62.5 (47, 73)	55.57 (42.25, 71)	62.37 (51.5, 74.5)	0.09
Gender, male, n (%)	77 (63.12)	38 (61.29)	39 (65)	0.81
Length of stay, median days (Q1, Q3)	16 (11, 28.75)	23 (13, 39)	13 (9, 20)	<0.001
Mortality rate, n (%)	7 (5.74)	6 (9.68)	1 (1.67)	0.11

^*^After Shapiro–Wilk tests, age and length of stay did not follow a normal distribution.

^#^The ICU group includes patients from the intensive care unit (ICU), emergency ICU, neurology ICU, coronary care unit, respiratory ICU, neonatal ICU, pediatric ICU, and those once admitted to the abovementioned units.

^**^The non-ICU group includes patients from the neurology department, neurosurgery department, respiratory medicine department, and hematology department.

^##^ Age and length of stay used Mann–Whiney U test, gender used Pearson’s χ^2^ test, and mortality rate used continuity correction Pearson’s χ^2^ test.

**Table 2:** Clinical conditions of patients.

Clinical conditions*, n (%)	Total (122)	ICU (62)	Non-ICU (60)	*p-*Value
Cerebral stroke^#^	45 (36.89)	39 (62.90)	4 (6.67)	<0.001
Coma	31 (25.41)	29 (46.77)	2 (3.33)	<0.001
Solid tumor	22 (18.03)	4 (6.62)	18 (30)	0.002
Diabetes	15 (12.30)	7 (11.29)	8 (13.33)	0.95
COPD**	12 (9.84)	2 (3.23)	10 (16.67)	0.03
Post-chemotherapy	11 (9.02)	0	11 (18.33)	0.001
Hematopoietic malignancies	9 (7.38)	0	9 (15)	0.001
Chronic kidney disease	9 (7.38)	5 (8.06)	4 (6.67)	1
Cardiovascular disease	7 (5.74)	4 (6.62)	3 (5)	1
Autoimmune diseases	5 (4.10)	1 (1.61)	4 (6.67)	0.20
Asthma	4 (3.28)	1 (1.61)	3 (5)	0.36
Bronchiectasis	3 (2.46)	0	3 (5)	0.12
Cardiac arrest	3 (2.46)	3 (4.84)	0	0.24

ICU, intensive care unit.

^*^One patient may have more than one condition.

^#^Traumatic intracranial hemorrhage was included.

^**^COPD, chronic obstructive pulmonary disease. Tests used either Pearson’s χ^2^ test or continuity correction Pearson’s χ^2^ test.

### Comparison of Pathogen Species Identified by Metagenomic Next-Generation Sequencing and Culture

**Figure 1:**
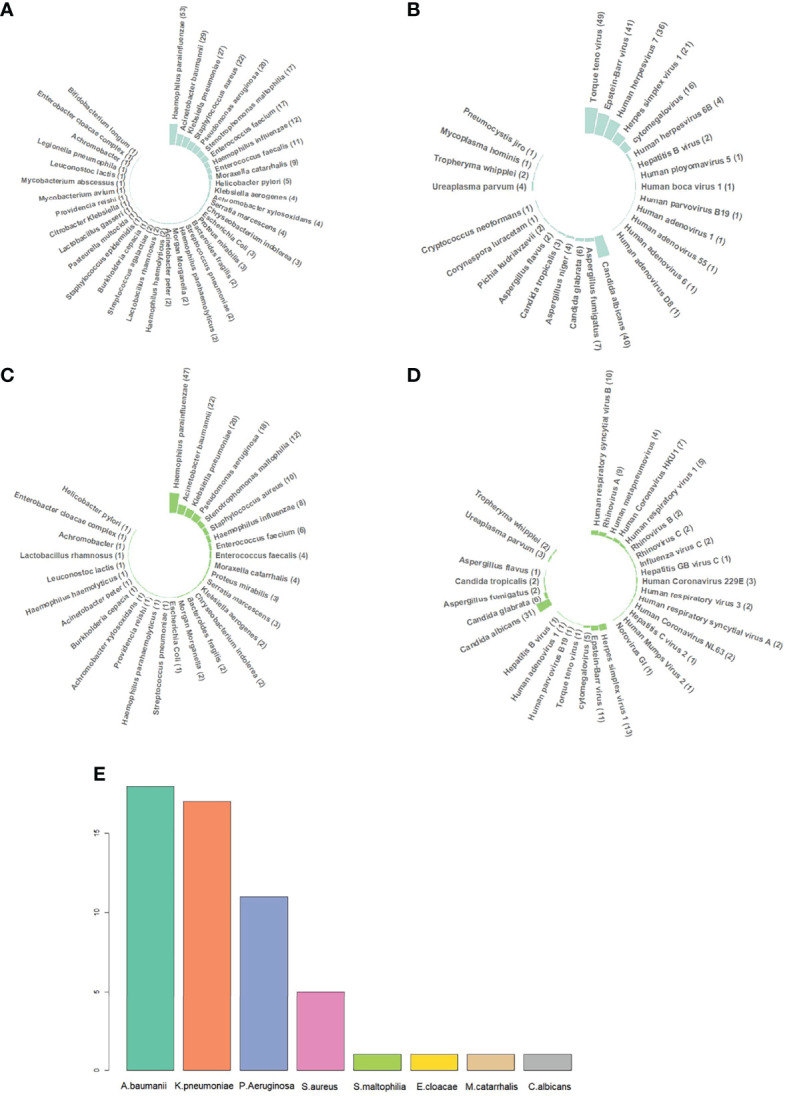
Different pathogen species identified by metagenomic next-generation sequencing (mNGS) and culture. **(A)** DNA sequencing results of bacteria. *Haemophilus parainfluenzae*, *Haemophilus influenzae*, *Enterococcus faecium*, *Enterococcus faecalis*, *Candida albicans*, etc., were commonly categorized into normal respiratory microbiomes, but they can still cause infection in immunocompromised patients. Therefore, all the sequencing results were listed, including those of respiratory microbiomes. **(B)** DNA sequencing results of DNA viruses, fungi, and atypical pathogens. **(C)** RNA sequencing results of bacteria. RNA sequencing was performed on 112 out of 122 samples. **(D)** RNA sequencing results of RNA viruses, DNA viruses, fungi, and atypical pathogens. **(E)** Sputum culture results. *H. parainfluenzae*, *H. influenzae*, *E. faecium*, *E. faecalis*, and *C. albicans* were categorized into normal respiratory microbiomes when cultured. If *H. influenzae* and *C. albicans* grew massively, they would be reported positive. Abbreviations: *A. baumannii*, *Acinetobacter baumannii*; *K. pneumoniae*, *Klebsiella pneumoniae*; *P. Aeruginosa*, *Pseudomonas aeruginosa*; *S. aureus*, *Staphylococcus aureus*; *S. maltophilia*, *Stenotrophomonas maltophilia*; *E. cloacae*, *Enterobacter cloacae*; *M. catarrhalis*, *Moraxella catarrhalis*; *C. albicans*, *Candida albicans*.

To identify the pathogens in sputum, DNA sequencing, RNA sequencing, and culture tests were performed on 121 (121/122, 99.18%), 112 (112/122, 91.80%), and 122 (122/122, 100%) samples, respectively. The results of mNGS and culture are shown in **Figure 1**. It should be noted that opportunistic pathogens were also listed in the results, such as *Haemophilus parainfluenzae*, *Haemophilus influenzae*, *Enterococcus faecium*, *Enterococcus faecalis*, and *Candida albicans*.

In DNA sequencing, the positive rate of pathogen detection reached 96.69% (117/121). A total of 272 strains of bacteria in 38 species, 66 strains of fungi in 9 species, and 176 viruses in 14 species or subspecies were detected, along with 8 strains of 4 atypical pathogens, including *Ureaplasma parvum* (4/121, 3.31%), *Mycoplasma hominis* (1/121, 0.82%), *Whipple whipplei* (2/121, 1.65%), and *Pneumocystis jirovecii* (1/121, 0.82%). The most abundant bacterial species was *H. parainfluenzae* (53/121, 43.80%), followed by *Acinetobacter baumannii* (29/121, 23.97%), *Klebsiella pneumoniae* (27/121, 22.31%), *Staphylococcus aureus* (22/121, 18.18%), *Pseudomonas aeruginosa* (20/121, 16.53%), *Stenotrophomonas maltophilia* (17/121, 14.05%), *E. faecium* (17/121, 14.05%), *H. influenzae* (12/121, 9.92%), *E. faecalis* (11/121, 9.10%), and *Moraxella catarrhalis* (9/121, 7.38%). The top 6 viruses were *Torque teno virus* (TTV) (49/121, 40.50%), *Epstein-Barr virus* (EBV) (41/121, 33.88%), *Human herpesvirus 7* (HHV-7) (36/121, 29.75%), *Herpes simplex virus 1* (HSV-1) (21/121, 17.36%), *Cytomegalovirus* (CMV) (16/121, 13.22%), and *Human herpesvirus 6B* (4/121, 3.31%). The top 5 fungi were *C. albicans* (40/121, 33.06%), *Aspergillus fumigatus* (7/121, 5.79%), *Candida glabrata* (6/121, 4.96%), *Aspergillus niger* (4/121, 3.31%), and *Candida tropicalis* (3/121, 2.48%).

In RNA sequencing, the positive rate of pathogen detection also reached 96.43% (108/112). All the bacteria, fungi, DNA viruses, and atypical pathogens detected by RNA sequencing had already been indicated in DNA sequencing. A total of 54 RNA viruses in 16 species or subspecies were detected, among which *Human respiratory syncytial virus B* was the most abundant species (10/112, 89.29%) followed by *Rhinovirus A* (9/112, 8.04%), *Human Coronavirus HKU1* (7/112, 6.25%), *Human respiratory virus 1* (5/112, 4.46%), *Human metapneumovirus* (4/112, 3.57%), and *Human Coronavirus 229E* (3/112, 2.68%). *Human Coronavirus NL63*, *Rhinovirus B*, *Rhinovirus C*, *Influenza virus C*, *Human respiratory virus 3*, and *Human respiratory syncytial virus A* accounted for 1.80% (2/112, 1.79%). *Hepatitis C virus 2*, *Human Mumps Virus 2*, and *Norovirus GI* were detected once only (1/112, 0.89%).

In culture tests, the positive rate of the pathogen was 39.34% (48/122), much lower than that of DNA and RNA sequencing. A total of 54 strains of bacteria in 7 species were detected, with *A. baumannii* being the most common one (18/122, 14.75%), followed by *K. pneumoniae* (17/122, 13.93%), *P. aeruginosa* (11/122, 9.02%), and *S. aureus* (5/122, 4.10%). *S. maltophilia*, *Enterobacter cloacae*, and *M. catarrhalis* were found once only (1/122, 0.82%). *C. albicans* was the only fungus identified from sputum culture because of its massive growth.

### Comparison of Overall Performance of Metagenomic Next-Generation Sequencing and Culture in Pathogen Detection

**Figure 2:**
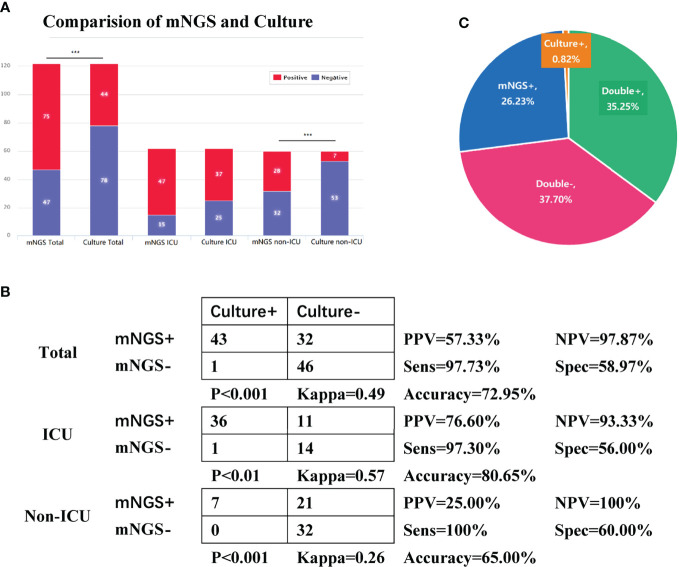
Comparison of overall performance of metagenomic next-generation sequencing (mNGS) and culture in pathogen detection. Tests used Pearson’s χ^2^ test. **(A)** The number of positive and negative cases in the Total, intensive care unit (ICU), and non-ICU groups of sputum mNGS and culture. **(B)** Fourfold contingency tables of the sensitivity and specificity of sputum mNGS and culture. We assumed sputum culture to be ground truth. Tests used McNemar’s chi-square test. **(C)** Pie chart of the positivity distribution of mNGS and culture. Abbreviations: PPV, positive predictive values; NPV, negative predictive values; Sens, sensitivity; Spec, specificity. Due to the limitations of culture-based methods in detecting certain opportunistic pathogens, fungi, and viruses, we decided to exclude *Haemophilus* spp., *Candida albicans*, *Enterococcus* spp., and viruses from the mNGS results before comparing the overall performance of these two methods in pathogen detection. Only one *C. albicans* was reported positive in sputum culture, and because of its massive growth, the corresponding mNGS result was listed. Since the bacteria and fungi in the RNA flow results were all included in the DNA flow, mNGS data were based on the data of the DNA flow.

Due to the limitations of culture-based methods in detecting certain opportunistic pathogens, fungi, and viruses, we decided to exclude *Haemophilus* spp., *C. albicans*, *Enterococcus* spp., and viruses from the mNGS results before comparing the overall performance of these two methods in pathogen detection. The positive rate of pathogen detection by sputum mNGS was significantly different from that of sputum culture in the total and non-ICU groups (p < 0.001) but did not show any significant difference when compared to the result of sputum culture in the ICU group (p = 0.08). In the Total group, the positive rate of pathogen detection by mNGS was 61.48% (75/122), ~25.41% higher than the 36.07% (44/122) of culture (**Figure 2A**). The PPV, NPV, and accuracy of diagnosing pulmonary infection in all cases by mNGS were 57.33%, 97.87%, and 72.95%, respectively. The positive likelihood ratio and negative likelihood ratio were 2.38 and 0.04, respectively.

In the total, ICU, and non-ICU groups, the sensitivity of mNGS was 97.73%, 97.3%, and 100%, respectively, showing that mNGS was of great value in diagnosing infection; the specificity of mNGS was 58.97%, 56%, and 60%, respectively. The sensitivity of mNGS was better than that of culture in all the three groups (p < 0.01), and the results were in good coincidence in the total and ICU groups (kappa = 0.49 and 0.57), while the consistency was average in the non-ICU group (kappa = 0.26) (**Figure 2B**). In general, sputum mNGS and culture were both positive in 43 of 122 (35.25%) cases and were both negative in 46 of 122 (37.7%) cases. There were 32 (26.23%) and 1 (0.82%) positive cases exclusive to mNGS and culture, respectively (**Figure 2C**). Only one mismatched case showed a difference in sputum culture and mNGS results. *Proteus mirabilis* was detected in mNGS, but *E. cloacae* was detected in the culture. Excluding the above case, all sputum culture results can be detected in the corresponding DNA testing procedure.

### Comparison of the Most Common Pathogens Detected by Metagenomic Next-Generation Sequencing Between the Intensive Care Unit and Non-Intensive Care Unit Groups

**Figure 3:**
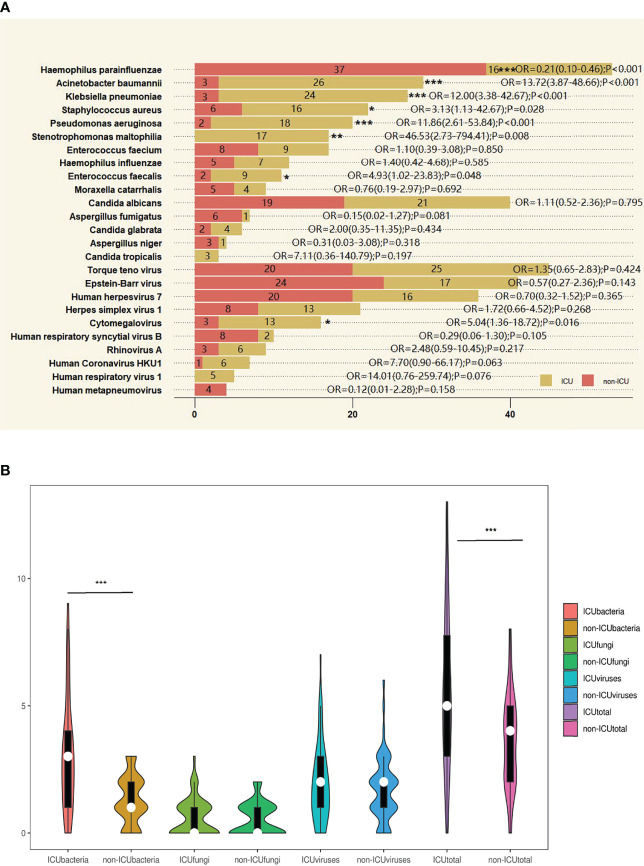
The overlap of metagenomic next-generation sequencing (mNGS) positivity between intensive care unit (ICU) and non-ICU groups for different pathogens. **(A)** The most common bacteria, fungi, DNA viruses, and RNA viruses were detected by mNGS method between ICU and non-ICU groups. The 10 most common bacteria and the 5 most common fungi, DNA viruses, and RNA viruses were detected by mNGS method between ICU and non-ICU groups. The last 5 viruses were RNA viruses detected by RNA sequencing. Ten ICU cases did not complete RNA sequencing. Other pathogens were detected by DNA sequencing. Tests used Z test. **(B)** Violin plot of bacteria, fungi, viruses, and all pathogens detected by DNA and RNA sequencing between ICU and non-ICU groups. After Shapiro–Wilk tests, they did not follow normal distribution; tests used Mann–Whiney U test. *p < 0.05, **p < 0.01, ***p < 0.001. OR, odds ratio.

Among the 10 most common bacteria implied from DNA sequencing results, the number of cases of some species showed significant differences between the ICU and non-ICU groups: *H. parainfluenzae*, *A. baumannii*, *K. pneumoniae*, and *P. aeruginosa* between groups (p < 0.001); *S. maltophilia* between groups (p < 0.01); and *S. aureus* and *E. faecalis* between groups (p < 0.05). In contrast, there were no significant differences between groups in terms of the number of cases of the 5 most common fungi identified by DNA sequencing, including *C. albicans*, *A. fumigatus*, *C. glabrata*, *A. niger*, and *C. tropicalis*. Likewise, the number of cases of the 5 most abundant DNA or RNA viruses in the ICU group was not significantly different from that in the non-ICU group, except for the DNA virus CMV (p < 0.05) (**Figure 3A**).

However, the number of pathogen species detected by mNGS varies greatly across all cases.

For the number of bacterial species detected in each case, the median was 3 (Q1, Q3, 1, 4) in the ICU group, significantly different from 1 (1, 2) in the non-ICU group (p < 0.001). With regard to the median number of fungi or viruses identified per case, there were no significant differences between groups. The median was 0 (0, 1) for fungi in both the ICU and non-ICU groups and 2 (1, 3) and 2 (1, 2) for viruses in the ICU group and the non-ICU group, respectively. When taking into account all pathogens including bacteria, fungi, viruses, and atypical pathogens, the median was 5 (3, 7.75) in the ICU group and 4 (2, 5) in the non-ICU group, showing a significant difference between groups (p < 0.001) (**Figure 3B**).

## Discussion

The traditional pathogen detection methods commonly used in clinical practice, such as culture, microscopy, and serological testing, tend to be time-consuming and have a low positive rate, making the search for efficient alternatives increasingly important to deliver a timely and accurate diagnosis (Miao et al., 2018; Wang et al., 2020). In infectious diseases like pulmonary infection, the pathogens may involve a broad class of microorganisms including bacteria, mycobacterium tuberculosis, viruses, fungi, mycoplasma, and chlamydia, the complexity of which poses further challenges for traditional methods in pathogen identification, especially in cases of mixed infections (Wang et al., 2019; Wu et al., 2020). In order to speed up the diagnosis process and guide clinicians to the most appropriate treatment, it is of crucial importance to look for efficient alternatives with a high degree of sensitivity and specificity, such as mNGS (Hong et al., 2020). With major progress on challenges (e.g., human DNA contamination) and continued decreases in cost, mNGS becomes an increasingly popular choice for clinical diagnosis (Ji et al., 2020).

With the help of mNGS, we aimed to explore the differences between pathogens in ICU and non-ICU patients with pulmonary infection, on the assumption that ICU patients are more susceptible to severe infections due to multiple traumas, cerebrovascular accidents, shock, immunosuppression, etc. Ideally, BALF is supposed to be used for pathogen detection in pulmonary infection. Nonetheless, BALF is not readily available in general wards, and thus, sputum was chosen instead.

For the comparisons made on patient attributes, there were no significant differences between the ICU group and the non-ICU group in age, gender, and mortality rate, except for the length of stay. In the ICU group, the most common clinical conditions were cerebral stroke and coma, while in the non-ICU group, the most common ones were solid tumor, COPD, post-chemotherapy, and hematopoietic malignancies. All these common conditions showed significant differences between groups, whereas diabetes and other conditions did not.

From the mNGS results, it could be seen that *H. parainfluenzae* was the most abundant bacterial species detected by DNA sequencing, accounting for 43.80% of all cases (53/121), and turned out to be more prevalent in the non-ICU group (p < 0.001). *H. parainfluenzae* is an opportunistic pathogen that has been associated with endocarditis, COPD, otitis media, and, in rare cases, brain abscesses (Pang and Swords, 2017; De Castro et al., 2019). As expected, nearly two-thirds of non-ICU patients with *H. parainfluenzae* infection suffered from tumors or COPD. When treated improperly, *H. parainfluenzae* may develop resistance mechanisms, that work alone or together, to confer decreased susceptibility to a variety of beta-lactam antibiotics (Garcia-Cobos et al., 2013). Although *H. parainfluenzae* was often considered a member of the normal flora, it may be the pathogen responsible for the disease in immunocompromised patients without other evidence of etiology, especially when antibiotics are not effective. The higher proportion of *H. parainfluenzae* in non-ICU patients is unclear, possibly because there are more patients with mixed infections and a higher proportion of broad-spectrum antibiotics in the ICU patients, resulting in a low proportion of patients detected in the ICU patients.

Unlike *H. parainfluenzae*, the next top 5 bacterial species detected by DNA sequencing, also the top 5 bacterial species identified by culture, appeared to be more common in the ICU group (p < 0.05), including *A. baumannii*, *K. pneumoniae*, *S. aureus*, *P. aeruginosa*, and *S. maltophilia*. Given the ability of *A. baumannii* to live on a variety of hospital surfaces such as surgical drains and catheters, the opportunistic pathogen *A. baumannii* is primarily associated with hospital-acquired infections, responsible for up to 2%–10% of all Gram-negative infections in the United States and Europe (Costa et al., 2006; Fournier et al., 2006). It is particularly common to find *A. baumannii* in ICU patients with susceptible immune systems and those who required endotracheal intubation or tracheostomy and invasive equipment such as catheters. Once infected, patients may develop a variety of diseases including pneumonia, meningitis, septicemia, and urinary and respiratory tract infections in spite of the fact that infection of *A. baumannii* is less common than colonization, and ventilator-associated pneumonia (VAP) and bloodstream infections have been reported to have high morbidity and mortality (Scerpella et al., 1995). In our study, most of the susceptibility tests demonstrated pan-antibiotic resistance in carbapenem-resistant *A. baumannii*. Similarly, *K. pneumoniae* is also an opportunistic pathogen, which has been implicated as one of the most common causes of hospital- and community-acquired infections involving urinary tract infections, pneumonia, and intraabdominal infections and has developed resistance against many common antibiotics, including tigecycline and carbapenem (Zhang et al., 2016). *K. pneumoniae* is becoming one of the top 8 pathogens in hospitals all around the world due to antibiotic resistance. According to the data from chinets.com, *K. pneumoniae* is the most common pathogen detected in respiratory specimens and is the second most common bacterium among all isolated strains (CHINET 2021). Interestingly, there was only one Gram-positive bacterium among the 6 most common bacteria identified by DNA sequencing, i.e., *S. aureus*. This bacterium is genetically close to its methicillin-resistant strain methicillin-resistant *S. aureus* (MRSA). Thus, the differentiation between these two strains could not be resolved by mNGS alone due to the limited genetic resources of *S. aureus* but relied more on antimicrobial susceptibility testing. *S. aureus* is among the most common hospital-acquired pathogens, which can cause bacteremia, pneumonia, and meningitis (Okwu et al., 2019). Compared with the abovementioned bacterial species, *P. aeruginosa* seems to be the “ubiquitous” one since it can live in both inanimate and human environments. This bacterial species tends to cause infections that are especially difficult to treat with its ability to form antibiotic-resistant biofilms. For example, VAP caused by *P. aeruginosa* is associated with attributable mortality rates of 40%, higher than the corresponding rates for most other causes of VAP (Hauser, 2008). The Centers for Disease Control and Prevention (CDC) has estimated the overall prevalence of *P. aeruginosa* infections in US hospitals at approximately 4 per 1,000 discharged patients (0.4%). *P. aeruginosa* accounts for 10.1% of all hospital-acquired infections, and it is the fourth most commonly isolated nosocomial pathogen. Based on the data from chinets.com, *P. aeruginosa* is the third most common pathogen detected in respiratory specimens and is the fifth most common bacterium among all isolated strains (CHINET 2021), similar to our results. Like other bacterial species, *S. maltophilia* is also an opportunistic pathogen causing an increasing number of nosocomial infections, especially in patients who are immunocompromised or those who require ventilatory support. Correspondingly, *S. maltophilia* was only found in ICU patients in this study.

For fungi identified by mNGS, the most abundant species was *C. albicans*, an opportunistic fungal pathogen that is responsible for candidiasis in human hosts. Typically, *C. albicans* resides as a harmless commensal in the gastrointestinal and genitourinary tract, found in over 70% of the population. Overgrowth of these organisms, however, will lead to disease, and it usually occurs in immunocompromised individuals, such as HIV-infected victims, transplant recipients, chemotherapy patients, and low-birth-weight babies (Kabir et al., 2012). Nevertheless, in this study, no differences were observed in *C. albicans* and other fungi between the ICU and non-ICU groups.

By DNA sequencing, mNGS uncovered the top 5 viruses in sputum: TTV, EBV, HHV-7, HSV-1, and CMV. TTV was the first *Anellovirus* with a circular single-stranded DNA genome identified in humans. *Anelloviruses* are very ubiquitous, representing about 70% of the viruses found within human blood and tissues (Freer et al., 2018). These viruses are associated with immune suppression and diseases that correlate with immune suppression, such as hepatitis, cancer, and autoimmune diseases (Blatter et al., 2018). The genetic variability and constant evolution of TTV contribute to its adaptation to new environments, and it may have been a part of humans. Whether or not TTV is pathogenic has yet to be discerned, but to date, there is little evidence for direct pathogenesis. EBV, formally called *Human gammaherpesvirus 4*, is a member of the *Lymphocryptovirus* genus, which belongs to the *Gammaherpesvirinae* subfamily of the *Herpesviridae* family. EBV-associated cancers consist of up to 15% of all human cancers involving a virus infection, but so far, there is limited progress in antivirals to deal with EBV infection (Amon and Farrell, 2005). HHV-7 is classified under the *Roseolovirus* genus of the *Betaherpesvirinae* subfamily, which can cause roseola infantum, a common disease of childhood developed by primary infection with HHV-6 and less frequently by HHV-7. After a primary infection, HHV-7 establishes a latent state and can be reactivated later, especially in hosts who are immunocompromised or with solid organ transplantation (Caserta et al., 1998). Likewise, immunocompromised patients are at an increased risk of developing severe HSV-1 infection, which may lead to extensive cutaneous or mucosal necrosis and even esophagitis or proctitis when the adjacent tissue is involved. HSV-1 can also lead to meningoencephalitis, pneumonia, hepatitis, and coagulation dysfunction (Berrington et al., 2009). The above four viruses had been detected in large quantities, but there were no differences between the ICU and non-ICU groups. Among the 5 most abundant DNA viruses, CMV was the only one showing a significant difference between groups. CMV is a common virus affecting ~50%–80% of the general adult population. In most cases, infected people are unaware of their infection since this virus is typically dormant in healthy individuals. CMV infection is also the most common viral infection among transplant patients, occurring in ~20%–60% of this cohort, and it can remain latent in multiple organs and even in bone marrow (Traylen et al., 2011). In immunosuppressed or post-transplant patients, CMV can lead to severe infections such as mixed bacterial/fungal pneumonia, retinitis, or even death.

By RNA sequencing, mNGS confirmed the presence of various RNA viruses in the sputum samples, including *Human respiratory syncytial virus* (RSV) B and A; *Rhinovirus* (RV) A, B, and C; *Human metapneumovirus* (HMPV); and *Human Coronavirus* (HCoV) HKU1, 229E, and NL63. However, all these RNA viruses did not show any significant differences between the ICU and non-ICU patients. RSV consists of two major subtypes, A and B. Subtype B is characterized as the asymptomatic strain, while subtype A is a major cause of more severe disease (Oliveira et al., 2008). RSV is considered the leading cause of lower respiratory tract infections characterized by bronchiolitis and pneumonia mainly in children, the elderly, and immunocompromised individuals. The severity of RSV infection is very diverse, ranging from mild cold symptoms to severe and life-threatening outcomes. For RV, it is best known for causing the common cold, although it has also been implicated in other clinical conditions such as bronchitis and asthma attacks. RV could very often be responsible for mild cold symptoms, including fever, cough, and runny nose (Cox and Le Souef, 2014). Along with RSV, HMPV is categorized into the *Paramyxoviridae* family, which can cause upper and lower respiratory infections in people of all ages, especially among young children, older adults, and immunocompromised individuals (Schildgen et al., 2011). Children under the age of five suffer more from pneumonitis and bronchiolitis, adults over the age of sixty-five tend to suffer from bronchitis and pneumonitis, and individuals with weakened immune systems suffer most from pneumonitis (Boivin et al., 2002). For HCoV-HKU1, HCoV-229E, and HCoV-NL63, these human coronaviruses are distributed globally among the human population. By spreading *via* coughing and sneezing, these viral strains generally cause mild upper respiratory tract diseases in adults but sometimes may cause life-threatening bronchiolitis and pneumonia in young children, the elderly, and immunocompromised individuals (Cabeca et al., 2013; Jain et al., 2015).

Overall, this study demonstrated the superiority of mNGS over culture in detecting all kinds of pathogen species in sputum samples, including bacteria, fungi, and viruses. Compared with sputum culture, mNGS showed a much higher value in the positive rate of pathogen detection, with the ability to identify up to 13 pathogens in one sample. Generally, the number of pathogen species detected by mNGS in each ICU patient was much higher than that in each non-ICU patient. These results indicate that mNGS may serve as a valuable tool to identify pathogens, especially for ICU patients who are more susceptible to mixed infections. Since RNA sequencing can detect not only RNA viruses but also the metabolic and replication activities of other pathogens, with the advancement of mNGS technology and the decline in detection costs, we can routinely perform RNA detection of sputum or BALF in the future.

Nonetheless, it should be noted that there were several limitations in this study. Due to the limited study funding, the number of patients in this study is relatively limited, and a larger sample size will be needed in the future for further validation. For mNGS, its performance in RNA virus detection among ICU patients might be compromised due to the lack of 10 RNA sequencing results. For the traditional method, it did not include the results of viral testing, since only a small proportion of patients had undergone traditional viral testing. In addition, it took nearly 15 days for all these samples to get tested and analyzed, and all mNGS results were not notified to clinicians to guide clinical treatment. Although mNGS has excellent pathogen detection ability, some questions are still inconclusive, such as which pathogens need targeted treatment, how to distinguish between infection and colonization, and whether the detected viruses need targeted antiviral treatment.

Given that infection is one of the leading causes of death in ICU patients, how to correctly interpret the results of the mNGS test, and how to avoid the abuse of antibiotics while choosing an anti-infective regimen, we need more large-scale research to solve these problems.

## Data Availability Statement

The original contributions presented in the study are publicly available. This data can be found here: NCBI Sequence Read Archive, Accession number SRR20851480, SRR20851481.

## Ethics Statement

The studies involving human participants were reviewed and approved by Medical Ethics Committee of the Affiliated Hospital of Xuzhou Medical University(XYFY2020-KL086-01). Written informed consent for participation was not required for this study in accordance with the national legislation and the institutional requirements.

## Author Contributions

J-JZ and Y-FC contributed substance to ideas and design. J-JZ and W-CD drafted the paper. LX and YY conducted statistical analysis on the data. Y-LG, Y-CL, and R-LG gave a lot of assistance and revises manuscript. All authors contributed to the article and approved the submitted version.

## Funding

This work was supported by the National Natural Science Foundation of China (No.81871593).

## Conflict of Interest

The authors declare that the research was conducted in the absence of any commercial or financial relationships that could be construed as a potential conflict of interest.

## Publisher’s Note

All claims expressed in this article are solely those of the authors and do not necessarily represent those of their affiliated organizations, or those of the publisher, the editors and the reviewers. Any product that may be evaluated in this article, or claim that may be made by its manufacturer, is not guaranteed or endorsed by the publisher.

## References

[B1] Amon W. Farrell P. J. (2005). Reactivation of Epstein-Barr Virus From Latency. Rev. Med. Virol. 15 (3), 149–156. doi: 10.1002/rmv.456 15546128

[B2] Berrington W. R. Jerome K. R. Cook L. Wald A. Corey L. Casper C. (2009). Clinical Correlates of Herpes Simplex Virus Viremia Among Hospitalized Adults. Clin. Infect. Dis. 49 (9), 1295–1301. doi: 10.1086/606053 19807272 PMC2803101

[B3] Blatter J. A. Sweet S. C. Conrad C. Danziger-Isakov L. A. Faro A. Goldfarb S. B.. (2018). Anellovirus Loads Are Associated With Outcomes in Pediatric Lung Transplantation. Pediatr. Transpl. 22 (1). doi: 10.1111/petr.13069 PMC5811341 29082660

[B4] Boivin G. Abed Y. Pelletier G. Ruel L. Moisan D. Cote S.. (2002). Virological Features and Clinical Manifestations Associated With Human Metapneumovirus: A New Paramyxovirus Responsible for Acute Respiratory-Tract Infections in All Age Groups. J. Infect. Dis. 186 (9), 1330–1334. doi: 10.1086/344319 12402203

[B5] Bolger A. M. Lohse M. Usadel B. (2014). Trimmomatic: A Flexible Trimmer for Illumina Sequence Data. Bioinformatics 30 (15), 2114–2120. doi: 10.1093/bioinformatics/btu170 24695404 PMC4103590

[B6] Cabeca T. K. Granato C. Bellei N. (2013). Epidemiological and Clinical Features of Human Coronavirus Infections Among Different Subsets of Patients. Influenza Other Respir. Viruses 7 (6), 1040–1047. doi: 10.1111/irv.12101 23462106 PMC4634278

[B7] Caserta M. T. Hall C. B. Schnabel K. Long C. E. D'Heron N. (1998). Primary Human Herpesvirus 7 Infection: A Comparison of Human Herpesvirus 7 and Human Herpesvirus 6 Infections in Children. J. Pediatr. 133 (3), 386–389. doi: 10.1016/s0022-3476(98)70275-6 9738722

[B8] Costa G. F. Tognim M. C. Cardoso C. L. Carrara-Marrone F. E. Garcia L. B. (2006). Preliminary Evaluation of Adherence on Abiotic and Cellular Surfaces of Acinetobacter Baumannii Strains Isolated From Catheter Tips. Braz. J. Infect. Dis. 10 (5), 346–351. doi: 10.1590/s1413-86702006000500009 17293924

[B9] Cox D. W. Le Souef P. N. (2014). Rhinovirus and the Developing Lung. Paediatr. Respir. Rev. 15 (3), 268–274. doi: 10.1016/j.prrv.2014.03.002 24767866

[B10] De Castro A. Abu-Hishmeh M. El H. I. Paul L. (2019). Haemophilus Parainfluenzae Endocarditis With Multiple Cerebral Emboli in a Pregnant Woman With Coronavirus. IDCases 18, e593. doi: 10.1016/j.idcr.2019.e00593 PMC6676006 31388489

[B11] Fiorini N. Lipman D. J. Lu Z. (2017). Towards PubMed 2.0. eLife 6. doi: 10.7554/eLife.28801 PMC5662282 29083299

[B12] Fournier P. E. Vallenet D. Barbe V. Audic S. Ogata H. Poirel L.. (2006). Comparative Genomics of Multidrug Resistance in Acinetobacter Baumannii. PloS Genet. 2 (1), e7. doi: 10.1371/journal.pgen.0020007 16415984 PMC1326220

[B13] Freer G. Maggi F. Pifferi M. Di Cicco M. E. Peroni D. G. Pistello M. (2018). The Virome and its Major Component, Anellovirus, a Convoluted System Molding Human Immune Defenses and Possibly Affecting the Development of Asthma and Respiratory Diseases in Childhood. Front. Microbiol. 9. doi: 10.3389/fmicb.2018.00686 PMC5902699 29692764

[B14] Garcia-Cobos S. Arroyo M. Campos J. Perez-Vazquez M. Aracil B. Cercenado E.. (2013). Novel Mechanisms of Resistance to Beta-Lactam Antibiotics in Haemophilus Parainfluenzae: Beta-Lactamase-Negative Ampicillin Resistance and Inhibitor-Resistant TEM Beta-Lactamases. J. Antimicrob. Chemother. 68 (5), 1054–1059. doi: 10.1093/jac/dks525 23335113

[B15] Geng S. Mei Q. Zhu C. Fang X. Yang T. Zhang L.. (2021). Metagenomic Next-Generation Sequencing Technology for Detection of Pathogens in Blood of Critically Ill Patients. Int. J. Infect. Dis. 103, 81–87. doi: 10.1016/j.ijid.2020.11.166 33227513

[B16] Gu W. Deng X. Lee M. Sucu Y. D. Arevalo S. Stryke D.. (2021). Rapid Pathogen Detection by Metagenomic Next-Generation Sequencing of Infected Body Fluids. Nat. Med. 27 (1), 115–124. doi: 10.1038/s41591-020-1105-z 33169017 PMC9020267

[B17] Gu W. Miller S. Chiu C. Y. (2019). Clinical Metagenomic Next-Generation Sequencing for Pathogen Detection. Annu. Rev. Pathol. 14, 319–338. doi: 10.1146/annurev-pathmechdis-012418-012751 30355154 PMC6345613

[B18] Hauser A. R. (2008). Pseudomonas Aeruginosa: An Uninvited Guest Refuses to Leave. Am. J. Respir. Crit. Care Med. 178 (5), 438–439. doi: 10.1164/rccm.200805-789ED 18713847

[B19] Hong N. Anh N. T. Mai N. Nghia H. Nhu L. Thanh T. T.. (2020). Performance of Metagenomic Next-Generation Sequencing for the Diagnosis of Viral Meningoencephalitis in a Resource-Limited Setting. Open Forum Infect. Dis. 7 (3), a46. doi: 10.1093/ofid/ofaa046 PMC7051036 32158774

[B20] Jain S. Self W. H. Wunderink R. G. Fakhran S. Balk R. Bramley A. M.. (2015). Community-Acquired Pneumonia Requiring Hospitalization Among U.S. Adults. N Engl. J. Med. 373 (5), 415–427. doi: 10.1056/NEJMoa1500245 26172429 PMC4728150

[B21] Ji X. C. Zhou L. F. Li C. Y. Shi Y. J. Wu M. L. Zhang Y.. (2020). Reduction of Human DNA Contamination in Clinical Cerebrospinal Fluid Specimens Improves the Sensitivity of Metagenomic Next-Generation Sequencing. J. Mol. Neurosci. 70 (5), 659–666. doi: 10.1007/s12031-019-01472-z 32002752

[B22] Johns Hopkins ABX Guide (2021) Johns Hopkins ABX Guide, Diagnosis and Treatment of Infectious Diseases. Available at: https://www.hopkinsguides.com/hopkins/index/Johns_Hopkins_ABX_Guide/All_Topics/ (Accessed March 20 2021).

[B23] Kabir M. A. Hussain M. A. Ahmad Z. (2012). Candida Albicans: A Model Organism for Studying Fungal Pathogens. ISRN Microbiol. 2012, 538694. doi: 10.5402/2012/538694 23762753 PMC3671685

[B24] Li Y. Sun B. Tang X. Liu Y. L. He H. Y. Li X. Y.. (2020). Application of Metagenomic Next-Generation Sequencing for Bronchoalveolar Lavage Diagnostics in Critically Ill Patients. Eur. J. Clin. Microbiol. Infect. Dis. 39 (2), 369–374. doi: 10.1007/s10096-019-03734-5 31813078 PMC7102353

[B25] Magill S. S. Edwards J. R. Bamberg W. Beldavs Z. G. Dumyati G. Kainer M. A.. (2014). Multistate Point-Prevalence Survey of Health Care-Associated Infections. N Engl. J. Med. 370 (13), 1198–1208. doi: 10.1056/NEJMoa1306801 24670166 PMC4648343

[B26] MCM, 12th Edition (2019) Manual of Clinical Microbiology. Available at: https://www.clinmicronow.org/doi/book/10.1128/9781683670438.MCM/ (Accessed March 15 2021).

[B27] Miao Q. Ma Y. Wang Q. Pan J. Zhang Y. Jin W.. (2018). Microbiological Diagnostic Performance of Metagenomic Next-Generation Sequencing When Applied to Clinical Practice. Clin. Infect. Dis. 67 (suppl_2), S231–S240. doi: 10.1093/cid/ciy693 30423048

[B28] Miller S. Naccache S. N. Samayoa E. Messacar K. Arevalo S. Federman S.. (2019). Laboratory Validation of a Clinical Metagenomic Sequencing Assay for Pathogen Detection in Cerebrospinal Fluid. Genome Res. 29 (5), 831–842. doi: 10.1101/gr.238170.118 30992304 PMC6499319

[B29] National Center for Biotechnology Information (2021) RefSeq: NCBI Reference Sequence Database. Available at: https://www.ncbi.nlm.nih.gov/refseq/ (Accessed March 13 2021).

[B30] Okwu M. U. Olley M. Akpoka A. O. Izevbuwa O. E. (2019). Methicillin-Resistant Staphylococcus Aureus (MRSA) and Anti-MRSA Activities of Extracts of Some Medicinal Plants: A Brief Review. AIMS Microbiol. 5 (2), 117–137. doi: 10.3934/microbiol.2019.2.117 31384707 PMC6642907

[B31] Oliveira T. F. Freitas G. R. Ribeiro L. Z. Yokosawa J. Siqueira M. M. Portes S. A.. (2008). Prevalence and Clinical Aspects of Respiratory Syncytial Virus a and B Groups in Children Seen at Hospital De Clinicas of Uberlandia, MG, Brazil. Mem Inst Oswaldo Cruz 103 (5), 417–422. doi: 10.1590/s0074-02762008000500002 18797752

[B32] Pang B. Swords W. E. (2017). Haemophilus Parainfluenzae Strain ATCC 33392 Forms Biofilms in Vitro and During Experimental Otitis Media Infections. Infect. Immun. 85 (9). doi: 10.1128/IAI.01070-16 PMC5563591 28674033

[B33] Scerpella E. G. Wanger A. R. Armitige L. Anderlini P. Ericsson C. D. (1995). Nosocomial Outbreak Caused by a Multiresistant Clone of Acinetobacter Baumannii: Results of the Case-Control and Molecular Epidemiologic Investigations. Infect. Control Hosp. Epidemiol. 16 (2), 92–97. doi: 10.1086/647063 7759825

[B34] Schildgen V. van den Hoogen B. Fouchier R. Tripp R. A. Alvarez R. Manoha C.. (2011). Human Metapneumovirus: Lessons Learned Over the First Decade. Clin. Microbiol. Rev. 24 (4), 734–754. doi: 10.1128/CMR.00015-11 21976607 PMC3194831

[B35] Schlaberg R. Chiu C. Y. Miller S. Procop G. W. Weinstock G. (2017). Validation of Metagenomic Next-Generation Sequencing Tests for Universal Pathogen Detection. Arch. Pathol. Lab. Med. 141 (6), 776–786. doi: 10.5858/arpa.2016-0539-RA 28169558

[B36] Traylen C. M. Patel H. R. Fondaw W. Mahatme S. Williams J. F. Walker L. R.. (2011). Virus Reactivation: A Panoramic View in Human Infections. Future Virol. 6 (4), 451–463. doi: 10.2217/fvl.11.21 21799704 PMC3142679

[B37] Wang J. Han Y. Feng J. (2019). Metagenomic Next-Generation Sequencing for Mixed Pulmonary Infection Diagnosis. BMC Pulm. Med. 19 (1), 252. doi: 10.1186/s12890-019-1022-4 31856779 PMC6921575

[B38] Wang C. Huang Z. Li W. Fang X. Zhang W. (2020). Can Metagenomic Next-Generation Sequencing Identify the Pathogens Responsible for Culture-Negative Prosthetic Joint Infection? BMC Infect. Dis. 20 (1), 253. doi: 10.1186/s12879-020-04955-2 32228597 PMC7106575

[B39] Wu X. Li Y. Zhang M. Li M. Zhang R. Lu X.. (2020). Etiology of Severe Community-Acquired Pneumonia in Adults Based on Metagenomic Next-Generation Sequencing: A Prospective Multicenter Study. Infect. Dis. Ther. 9 (4), 1003–1015. doi: 10.1007/s40121-020-00353-y 33170499 PMC7652912

[B40] Zhang R. Lin D. Chan E. W. Gu D. Chen G. X. Chen S. (2016). Emergence of Carbapenem-Resistant Serotype K1 Hypervirulent Klebsiella Pneumoniae Strains in China. Antimicrob. Agents Chemother. 60 (1), 709–711. doi: 10.1128/AAC.02173-15 26574010 PMC4704206

